# The mediating role of sacral slope in the relationship between lumbar lordosis and knee alignment in knee osteoarthritis: an imaging study

**DOI:** 10.3389/fbioe.2026.1760345

**Published:** 2026-02-20

**Authors:** Binghua Zhang, Youyue Pang, Yicong Bai, Shilin Yin, Yuntao Yan, Xi Li, Xiang Wang, Yongwang Zhang, Chang Wang, Shuangqing Du

**Affiliations:** 1 Hebei Provincial Hospital of Chinese Medicine, Shijiazhuang, China; 2 Department of Orthopaedics, Hebei Provincial Hospital of Chinese Medicine, Shijiazhuang, China; 3 Hebei Key Laboratory of Turbidity Toxin Syndrome, Shijiazhuang, China

**Keywords:** imaging study, knee osteoarthritis, mediation, sacral slope, sagittal force line

## Abstract

**Objective:**

This study aimed to investigate the association between lumbar lordosis (LL) and the sagittal hip-knee-ankle angle (sHKA) in patients with knee osteoarthritis (KOA) and to quantify the mediating effect of the sacral slope (SS).

**Methods:**

This study enrolled 507 participants (left-side 302, right-side 205). Lateral full-length X-ray films of the lower extremities in weight-bearing position were collected from the research participants to measure radiological parameters such as lumbar lordosis (LL), sacral slope (SS), and sagittal hip-knee-ankle angle (sHKA), and SF-12 (12-items Short Form Health Survey) and WOMAC (Western Ontario and McMaster Universities Osteoarthritis Index) scores were also collected. Correlation analysis and Bootstrap mediation effect analysis were performed.

**Results:**

Among the 507 KOA participants, LL was positively correlated with SS, HKA, sHKA, and SF-12 scores, and negatively correlated with JLCA and WOMAC scores. The mediation analysis revealed that SS accounted for 10.74% of the total association of LL on sHKA.

**Conclusion:**

In patients with KOA, LL is closely related to sHKA, and this statistical association may be partially mediated by SS. This highlights the importance of adopting a “spine-pelvis-knee” perspective when assessing and treating KOA.

## 1 Introduction

Knee osteoarthritis (KOA) is a degenerative joint disease characterized by the degeneration of articular cartilage, synovial inflammation, and abnormal remodeling of subchondral bone (Barnett, 2018). It ranks as the fourth leading cause of disability worldwide (Safiri et al., 2020). Abnormal alterations in knee joint loading contribute significantly to the onset and progression of KOA.

During standing and walking, the spine, pelvis, and lower limbs coordinate to maintain postural balance. In KOA patients, the sagittal alignment of the spinopelvic-lower extremity kinetic chain undergoes a series of adaptive changes. Knee flexion contracture due to KOA leads to functional limitation and an anterior shift in the center of gravity. To compensate and maintain balance, the body initiates mechanisms such as decreased lumbar lordosis and pelvic retroversion (Lafage et al., 2009; Hambrecht et al., 2025). As the condition progresses, increased thoracic kyphosis may develop, requiring further compensation through additional pelvic retroversion and knee flexion (Fu et al., 2023; Katsumi et al., 2023; Tomay Aksoy and Bütün, 2025). Initial knee joint dysfunction triggers structural compensation in the upstream (spinal-pelvis), and these compensatory postures in turn increase the abnormal stress load in the downstream (knee joint), thus forming a vicious cycle of “knee joint dysfunction - spinal imbalance” with the pelvis as the hub, which ultimately accelerates joint degeneration (Murata et al., 2003; Le Huec et al., 2011; Iyer et al., 2018).

Although existing evidence indicates associations between spinal sagittal parameters and knee alignment in KOA patients, the mediating role of sacral slope in this relationship remains unclear. Therefore, we hypothesized that there would be a correlation between lumbar lordosis and abnormal sagittal knee alignment as reflected by sacral slope. This study aimed to: (1) verify the associations between the lumbar spine, pelvis, and knee joint, and (2) quantify the mediating effect of sacral slope, thereby providing a new perspective for establishing an integrated spine-pelvis-knee diagnosis and treatment strategy for KOA.

## 2 Patients and methods

### 2.1 Study participants and selection

**FIGURE 1:**
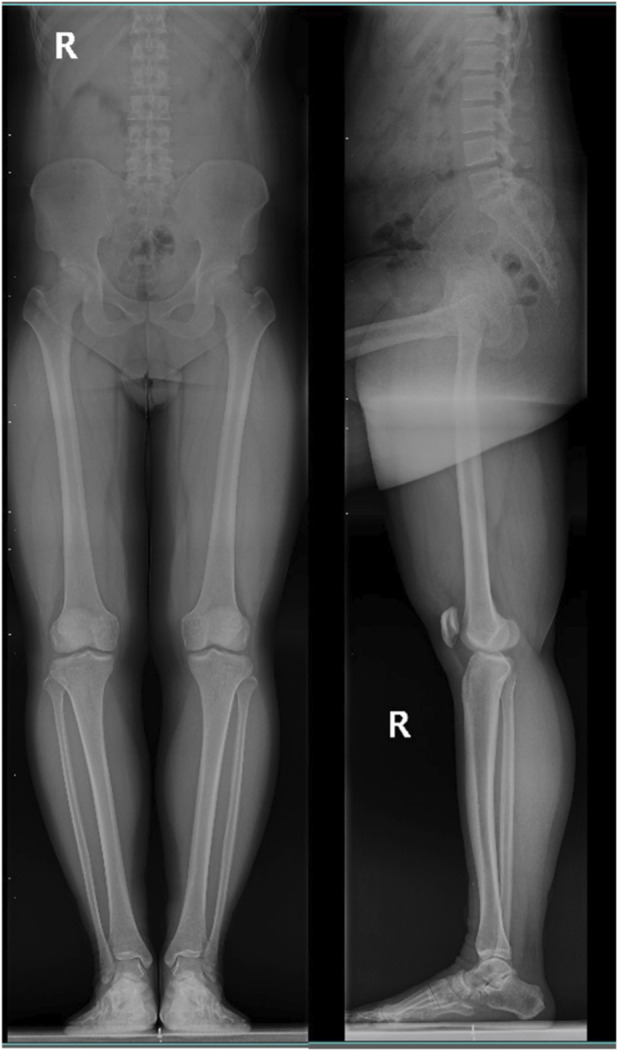
Radiographic projection.

This prospective study recruited participants from the Orthopaedics outpatient clinic of Hebei Provincial Hospital of Traditional Chinese Medicine. Participants who met all inclusion criteria and none of the exclusion criteria were enrolled. Ultimately, demographic information, WOMAC scores, and SF-12 scores were collected from 507 participants, and full-length lower limb radiographs including the lumbar spine were obtained (using the right side as an example, Figure 1).

### 2.2 Inclusion criteria

Participants diagnosed with KOA (unilateral or bilateral), Kellgren-Lawrence (K-L) grade II–IV, aged 50–85 years, either sex, voluntary participation with signed informed consent.

### 2.3 Exclusion criteria

History of compressive fractures from L1 to S1, spondylolisthesis, or lumbar spine surgery, combined with severe osteoporosis (axial bone T-score ≤ −2.5 SD by DXA), knee joint tumors, tuberculosis, or other diseases, knee arthroscopy or knee injury within the past year, or history of knee replacement surgery, inability to cooperate with the study procedures or follow-up, combined with severe primary cardiovascular or cerebrovascular diseases, mental illness, or malignant tumors, use of corticosteroids or hyaluronates within 3 months.

Demographic characteristics of the participants were uniformly collected. Height and weight were measured to the nearest 0.1 cm and 0.1 kg, respectively, and converted to body mass index (BMI: kg/m^2^). All 507 participants completed the questionnaires and weight-bearing full-length anteroposterior and lateral radiographs including the lumbar spine. The design and reporting of this study adhered to the Strengthening the Reporting of Observational Studies in Epidemiology (STROBE) guidelines. The study was approved by the Institutional Review Board of Hebei Provincial Hospital of Traditional Chinese Medicine (No. CE22468B) and strictly followed the principles of the Declaration of Helsinki. All participants provided voluntary written informed consent.

### 2.4 Patient-reported outcome measures

Patient-reported outcome measures (PROMs) were collected, including SF-12 and WOMAC. Higher SF-12 scores indicate more favorable outcomes, while higher WOMAC scores indicate worse outcomes.

### 2.5 Radiographic measurements

To obtain the lateral X-ray photos of lower limbs including lumbar vertebrae, standing on one leg. All images were obtained by the same technician using the same machine (Optima XR646 HD). After automated measurement of lumbar lordosis and the affected knee’s mechanical axis angles by the machine, two project team members reviewed the results.

Lumbar Lordosis (LL): The angle between the superior endplate of L1 and the inferior endplate of L5 on the sagittal view.

Sacral Slope (SS): The angle between the sacral plate and the horizontal line.

Pelvic Incidence (PI): The angle between the line perpendicular to the sacral plate at its midpoint and the line connecting the sacral plate midpoint to the midpoint of the bilateral femoral heads.

Hip-Knee-Ankle Angle (HKA): The angle formed by the center of the femoral head, the center of the knee, and the center of the ankle.

Sagittal Hip-Knee-Ankle Angle (sHKA): The angle formed by the sagittal center of the femoral head, the sagittal center of the knee, and the sagittal center of the ankle.

**FIGURE 2:**
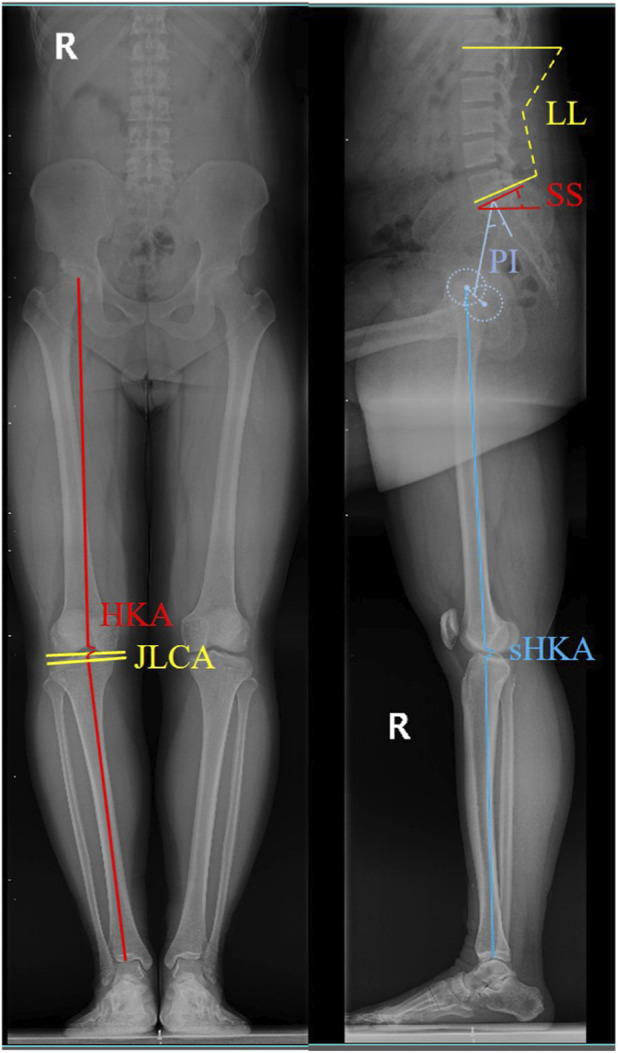
Joint angle measurement methods.

Joint-Line Convergence Angle (JLCA): The angle between the distal femur and the proximal tibia (Figure 2).

### 2.6 Statistical analysis

Spearman’s rank correlation coefficient was used to assess the associations between LL, SS, HKA/sHKA, and patient-reported outcomes (WOMAC). The mediation effect of SS was analyzed using Baron and Kenny’s causal steps approach combined with Bootstrapping (5,000 samples). Continuous variables are presented as mean ± standard deviation (SD), and categorical variables as frequencies. A P-value <0.05 was considered statistically significant. All statistical analyses were performed using SPSS version 25.0 (IBM Corp, United States).

## 3 Results

### 3.1 Baseline characteristics of the study participants

A total of 507 participants met the inclusion criteria (Table 1). The study included 109 males (21.5%) and 398 females (78.5%), age was 63.3 ± 8.2 years (50–85), BMI was 25.9 ± 3.1 kg/m^2^ (17–39), Bone Density was −1.4 ± 1.1 (−5–3.3). The WOMAC score was 40.31 ± 13.41 (9.0–82.0), and the SF-12 score was 38.86 ± 17.53 (6.6–89.6). The LL was 36.94 ± 8.79 (20.1–63.7), SS was 33.51 ± 8.67 (15.1–49.8), HKA was 176.08 ± 2.84 (169.1–181.0), sHKA was 175.20 ± 2.98 (169.0–180.8), JLCA was 2.25 ± 1.36 (0.0–6.0), PI was 53.13 ± 10.39 (37.0–83.7).

**TABLE 1:** Base line.

Group	Analysis group	Total	%
Gender	Female	398	78.5
	Male	109	21.5
KL	2	200	39.4
	3	225	44.4
	4	82	16.2

Total (N = 507)	Minimum	Maximum	Average	SD
Age	50	85	63.3	8.2
BMI	17	39	25.9	3.1
Bone density	−5	3.3	−1.4	1.1
WOMAC	9.0	82.0	40.31	13.41
SF-12	6.6	89.6	38.86	17.53

Group	Minimum	Maximum	Average	SD
LL	20.1	63.7	36.94	8.79
SS	15.1	49.8	33.51	8.67
HKA	169.1	181.0	176.08	2.84
sHKA	169.0	180.8	175.20	2.98
JLCA	0.0	6.0	2.25	1.36
PI	37.0	83.7	53.13	10.39
WOMAC	9.0	82.0	40.31	13.41
SF-12	6.6	89.6	38.86	17.53

Abbreviations: KL, Kellgren-Lawrence; SD, standard deviation; BMI, body mass index; WOMAC, Western Ontario and McMaster Universities Osteoarthritis Index, SF-12, Short Form-12 Health Survey; LL, lumbar lordosis; SS, sacral slope; HKA, Hip-Knee-Ankle, sHKA, sagittal Hip-Knee-Ankle, JLCA, joint line convergence angle; PI, pelvic incidence.

### 3.2 Correlation analysis between joint angles and scores

Spearman correlation analysis of joint angles for all 507 participants (Table 2) showed that LL was positively correlated with SS (r = 0.339, P < 0.01), HKA (r = 0.224, P < 0.01), sHKA (r = 0.498, P < 0.01), and SF-12 scores (r = 0.162, P < 0.01), but not significantly correlated with PI (r = 0.004, P > 0.05). LL was negatively correlated with JLCA (r = −0.314, P < 0.01), and WOMAC scores (r = −0.135, P < 0.01).

**TABLE 2:** Correlation test between joint angle and score.

Variable	SS	HKA	sHKA	JLCA	WOMAC	SF-12
LL	0.339**	0.224**	0.498**	−0.314**	−0.135**	0.162**

Abbreviations, LL, lumbar lordosis; SS, sacral slope; HKA, Hip-Knee-Ankle, sHKA, sagittal Hip-Knee-Ankle; JLCA, joint line convergence angle; WOMAC, Western Ontario and McMaster Universities Osteoarthritis Index; SF-12, Short Form-12 Health Survey. *P < 0.05, **P < 0.01, ***P < 0.001, n = 507.

### 3.3 Quantification of the mediating effect of SS

The mediation analysis revealed a significant positive association between SS and LL (β = 0.389, bootstrap 95% CI: 0.298–0.469, p < 0.01). No significant associations were found for the other factors (Table 3). In another model, significant positive associations with sHKA were found for LL (β = 0.149, bootstrap 95% CI: 0.124–0.181, p < 0.01) and SS (β = 0.137, bootstrap 95% CI: 0.020–0.075, p < 0.01). No significant associations were observed for the remaining factors (Table 3). Overall, in this statistical analysis, the direct effect of LL on sHKA accounted for 89.26% of the total effect, while the mediating effect of SS accounted for 10.74%. This suggests that SS plays a limited explanatory role in the association between LL and sHKA, and other factors may also be involved. After further incorporating WOMAC and SF-12, the intermediary structure of LL-SS-sHKA is consistent in direction and scale. The positive correlation between LL and SS remains stable, and the effect quantity fluctuates slightly but not significantly (Supplementary Table S1). After stratification by gender, the statistical analysis indicated that the mediating effect of SS was not significant in males (β = 0.052, bootstrap 95% CI: −0.011–0.164, p < 0.01) but was significant in females (β = 0.055, bootstrap 95% CI: 0.017–0.097, p < 0.01). The indirect effect accounted for 10.46% of the total effect (Table 4).

**TABLE 3:** Intermediary analysis of SS Bootstrap5000.

Variable	SS					sHKA				
	Coeff	se	LLCI	ULCI	p	Coeff	se	LLCI	ULCI	p
LL	0.389	0.043	0.298	0.469	<0.01	0.149	0.015	0.124	0.181	<0.01
SS	​	​	​	​	​	0.138	0.014	0.020	0.075	<0.01
Gender	−0.024	0.871	−2.225	1.196	0.554	0.020	0.275	−0.394	0.684	0.597
Age	0.032	0.042	−0.051	0.115	0.451	−0.036	0.013	−0.039	0.014	0.352
KL	0.025	0.519	−0.720	1.318	0.564	−0.004	0.164	−0.338	0.305	0.920
BMI	−0.009	0.118	−0.257	0.208	0.835	−0.030	0.037	−0.102	0.044	0.432
Bone density	0.057	0.337	−0.217	1.107	0.187	−0.068	0.106	−0.393	0.025	0.848
VAS	−0.050	0.233	−0.738	0.176	0.227	0.013	0.073	−0.119	0.170	0.729
JLCA	−0.031	0.278	−0.742	0.349	0.478	0.001	0.088	−0.169	0.175	0.971
PI	0.035	0.035	−0.039	0.098	0.401	−0.118	0.011	−0.056	−0.012	0.002
Total effect	​	​	​	​	​	0.503	0.014	0.143	0.198	<0.01
Direct effect	​	​	​	​	​	0.449	0.015	0.124	0.181	<0.01
Indirect effect	​	​	​	​	​	0.054	0.018	0.021	0.090	​

Abbreviations, BMI, body mass index; VAS, visual analogue scale; KL, Kellgren-Lawrence; LL, lumbar lordosis; SS, sacral slope; sHKA, sagittal Hip-Knee-Ankle; JLCA, Joint-Line Convergence Angle; PI, pelvic incidence; se, Standard error; LLCI, lower level of confidence interval; ULCI, upper level of confidence interval.

**TABLE 4:** Hierarchical analysis based on gender.

Dependent variable: SS	Male					Female				
	Coeff	se	LLCI	ULCI	p	Coeff	se	LLCI	ULCI	p
LL	0.418	0.104	0.212	0.624	<0.01	0.386	0.048	0.291	0.481	<0.01
Age	0.048	0.076	-0.103	0.199	0.528	0.044	0.052	-0.058	0.146	0.396
KL	2.183	1.091	0.019	4.347	0.048	-0.182	0.592	-1.346	0.983	0.759
BMI	0.240	0.252	-0.260	0.740	0.343	-0.078	0.136	-0.346	0.190	0.568
Bone density	-0.995	0.737	-2.457	0.466	0.180	0.781	0.385	0.024	1.538	0.043
VAS	-0.360	0.545	-1.442	0.721	0.510	-0.310	0.261	-0.824	0.204	0.237
JLCA	-0.383	0.650	-1.673	0.908	0.557	-0.153	0.310	-0.763	0.456	0.621
PI	0.039	0.072	-0.103	0.182	0.583	0.032	0.041	-0.048	0.111	0.437

Dependent variable: sHKA	Coeff	se	LLCI	ULCI	p	Coeff	se	LLCI	ULCI	p
LL	0.146	0.039	0.068	0.224	<0.01	0.155	0.016	0.124	0.186	<0.01
SS	0.049	0.035	-0.021	0.119	0.170	0.047	0.016	0.016	0.077	<0.01
Age	-0.011	0.027	-0.064	0.042	0.673	-0.008	0.016	-0.039	0.024	0.626
KL	-0.499	0.390	-1.273	0.275	0.204	0.081	0.182	-0.277	0.438	0.657
BMI	-0.051	0.089	-0.227	0.125	0.568	-0.030	0.042	-0.113	0.052	0.469
Bone density	-0.631	0.261	-1.149	-0.114	0.017	-0.088	0.119	-0.321	0.146	0.461
VAS	-0.080	0.192	-0.460	0.301	0.678	0.046	0.080	-0.112	0.204	0.566
JLCA	-0.376	0.229	-0.829	0.078	0.103	0.076	0.095	-0.111	0.263	0.424
PI	-0.025	0.025	-0.075	0.025	0.330	-0.061	0.102	-0.260	0.139	0.551
Total effect	0.425	0.037	0.094	0.239	<0.01	0.526	0.015	0.144	0.202	<0.01
Direct effect	0.373	0.039	0.068	0.224	<0.01	0.472	0.016	0.124	0.186	<0.01
Indirect effect	0.052	0.045	-0.011	0.164		0.055	0.020	0.017	0.097	

Abbreviations: BMI, body mass index; VAS, visual analogue scale; KL, Kellgren-Lawrence; LL, lumbar lordosis; SS, sacral slope; sHKA, sagittal Hip-Knee-Ankle; JLCA, Joint-Line Convergence Angle; PI, pelvic incidence; se, Standard error; LLCI, lower level of confidence interval; ULCI, upper level of confidence interval.

## 4 Discussion

This study investigated the interactions within the sagittal spinopelvic-knee alignment in KOA patients. Our main findings can be summarized as follows: First, correlation analysis confirmed significant, albeit varying in strength, associations between LL, SS, HKA, and patient-reported outcomes. Second, while SS accounted for 10.74% of the total effect between LL and sHKA and this finding was statistically significant. However, the cross-sectional study design can’t determine the causal relationship. Finally, the direct effect of LL on sHKA is dominant in statistical analysis. These findings provide a statistical basis for understanding the biomechanical mechanism of “spine-pelvis-knee” movement chain imbalance in KOA progress, and are a methodological refinement of previous theoretical research on spine-knee.

### 4.1 The vicious cycle of sagittal imbalance in KOA

This study confirmed the statistical associations between LL, SS, and sHKA in KOA patients, consistent with the previously proposed “spine-pelvis-knee kinetic chain” theory. KOA leads to knee flexion contracture, which subsequently affects the spine and pelvis, altering overall sagittal balance (Katsumi et al., 2023). In KOA patients, knee flexion contracture causes an anterior shift in the center of gravity. Compensatory adjustments, including decreased lumbar lordosis (reduced LL) and pelvic retroversion (reduced SS), are initiated to maintain sagittal balance in the short term. However, these compensations may exacerbate knee degeneration through two potential pathways: pelvic retroversion increases quadriceps tension and patellofemoral joint pressure, leading to increased local stress and aggravated knee symptoms (Otsuki et al., 2021), decreased lumbar lordosis reduces the dynamic stabilizing effect of paraspinal muscles on the spine, resulting in long-term accumulation of abnormal load on the lower limb alignment (Nakanishi et al., 2025). Sagittal spinal misalignment is associated with knee pain in KOA patients (Nakanishi et al., 2025), a conclusion supported by the negative correlation between LL and WOMAC scores found in our study.

Specifically, our study verified significant positive correlations between LL and SS (r = 0.339, p < 0.01) and between LL and sHKA (r = 0.498, p < 0.01), aligning with the spinopelvic-lower extremity overall kinetic chain theory (Murata et al., 2003; Oshima et al., 2019). A key finding is that the association strength between LL and sHKA (r = 0.498) was markedly higher than that between LL and the coronal HKA (r = 0.224), suggesting that sagittal alignment parameters might hold greater biomechanical significance than traditional coronal parameters in KOA-related sagittal imbalance. Furthermore, the negative correlation between LL and WOMAC scores (r = −0.135, p < 0.05) and the positive correlation with SF-12 scores (r = 0.162, p < 0.05) indicate that the reduction of lumbar lordosis may be related not only to more serious knee joint symptoms, but also to worse overall health. This supports clinical observations that knee flexion contracture due to KOA triggers an anterior shift in the center of gravity, subsequently inducing decreased lumbar lordosis and increased pelvic retroversion. While compensating posture short-term, these changes potentially alter lower limb mechanics, increase localized joint stress, and accelerate joint degeneration, forming a vicious cycle.

### 4.2 The mediating role of SS in sagittal imbalance and clinical implications

In patients with KOA, the process of knee flexion and mechanical axis imbalance primarily involves pelvic retroversion in the early stages to maintain overall balance and horizontal gaze, while later stages require combined compensatory mechanisms, such as reduced LL, to further elevate the center of gravity and compensate for sagittal plane imbalance (Tsuji et al., 2002; Wang et al., 2016; Hasegawa et al., 2017; Erp et al., 2020; Iwamura et al., 2020). This conclusion aligns with the significant correlations we observed among LL, sacral slope SS, and sHKA in KOA patients. The synchronous changes in LL, SS, and sHKA further suggest that SS may act as an intermediate link in the spine-lower limb kinetic chain (Wang et al., 2016). Therefore, based on the significant correlations among SS, LL, and sHKA, and to further explore the role of the pelvis, a Bootstrap mediation analysis framework was employed to examine whether SS could explain part of the statistical pathway connecting LL and sHKA. The results showed a significant positive correlation between LL and SS. After including SS, the association between LL and sHKA remained significant, but the effect size decreased, indicating that SS may account for part of the statistical pathway between LL and sHKA, with the indirect effect constituting approximately 10.74% of the total effect. After adjusting for confounding factors, the mediation structure of LL-SS-sHKA remained consistent in both direction and magnitude of effect. The positive correlation between LL and SS remained stable, with limited changes in effect size, and the results remained robust. Gender-stratified analysis revealed that the LL-SS-sHKA mediation structure was stronger in females and did not reach significance in males. The reasons for this finding warrant further investigation. On the one hand, due to the differing incidence of KOA between males and females (Szilagyi et al., 2023), the gender ratio of participants in this study was imbalanced, with a smaller number of males, which may have led to insufficient statistical power. Additionally, some studies have indicated that gender is a key factor influencing LL, with different LL levels observed between males and females (Miranda et al., 2022; Grabara and Witkowska, 2024). These factors may all influence the statistical results; therefore, this finding may not represent a true gender difference. Future studies need to further validate potential gender heterogeneity in larger, gender-balanced samples. It is important to emphasize that the mediation analysis employed in this study is based on cross-sectional data, which reveals statistical associations and indirect effects among variables conforming to the mediation model, but cannot establish the direction or mechanisms of causality between the variables.

### 4.3 Discussion of possible mechanisms

The interplay between knee flexion deformity, pelvic retroversion, and decreased lumbar lordosis represents a significant biomechanical covariation pattern, often referred to as LL-SS-sHKA, which profoundly influences sagittal spinal alignment and overall postur (Cheng et al., 2017; Perrier et al., 2023). This complex relationship involves compensatory mechanisms whereby changes in one segment of the kinetic chain—particularly the lower extremities—trigger adaptive responses in the pelvis and spine to maintain an upright stance and balance (Lee et al., 2013; Shimizu et al., 2020). It is particularly important to consider that knee flexion deformity may act as a driving factor, simultaneously influencing pelvic retroversion (manifested as reduced SS) and decreased lumbar lordosis, thereby forming the observed covariation pattern of LL-SS-sHKA (Pizones and García-Rey, 2020; Yucekul et al., 2023; Mohanty et al., 2024). This potential compensatory chain highlights the significance of knee joint status within the holistic spine-pelvis-lower limb biomechanics.

### 4.4 Clinical significance

The clinical significance lies in the need to adopt a holistic perspective when assessing KOA patients, incorporating compensatory changes in lumbar lordosis and sacral slope into consideration. Although causal relationships remain unconfirmed, identifying these compensatory features that co-occur within the spine-pelvis-lower limb chain and implementing integrated management strategies targeting the lumbar-pelvic-knee complex may help improve clinical evaluation and treatment planning, with the aim of achieving better functional outcomes.

### 4.5 Limitations and future directions

This study has several limitations. First, its cross-sectional design cannot establish causal relationships. Second, reliance on static, two-dimensional radiographic measurements may not fully capture three-dimensional dynamic biomechanics. Third, the high proportion of female participants may affect the generalizability of the findings. Finally, although significant, the mediating effect of the pelvis (10.74%) was relatively small, suggesting the existence of other important pathways. Future longitudinal studies and three-dimensional dynamic analyses are needed for further validation.

Future research should aim to address these limitations through longitudinal cohorts, 3D imaging (e.g., EOS imaging system), inclusion of more comprehensive spinopelvic and lower-limb parameters, and integration of dynamic motion analysis. Investigating the effects of targeted interventions (e.g., lumbar-pelvic rehabilitation) on this kinetic chain would be crucial for translating these findings into clinical practice. Future longitudinal or interventional studies are essential to elucidate the complex bidirectional or common causal relationships among these factors.

## 5 Conclusion

In this study, the intermediary analysis is innovatively applied to the dynamic chain of spine-pelvis-knee joint, which provides statistical basis for the intermediary role of sacral inclination in lumbar lordosis and sagittal position of knee joint. This not only provides direct data evidence for the connection theory of “spine-pelvis-knee”, but also provides a new perspective for the comprehensive biomechanical evaluation of knee osteoarthritis and understanding the compensation mechanism of sports chain.
