# Supplementary material for: The mediating role of sacral slope in the relationship between lumbar lordosis and knee alignment in knee osteoarthritis: an imaging study
Source: Front Bioeng Biotechnol. 2026 Feb 20;14:1760345. doi: 10.3389/fbioe.2026.1760345 (PMC12963331; doi:10.3389/fbioe.2026.1760345)
Supplement: Supplementary file 1 [file Table1.docx]

**Table S1** Intermediary analysis of SS Bootstrap5000 confounder adjustment

| Variable | SS | | | |  | | sHKA | | | |  |
| --- | --- | --- | --- | --- | --- | --- | --- | --- | --- | --- | --- |
|  | Coeff | se | LLCI | ULCI | p | | Coeff | se | LLCI | ULCI | p |
| LL | 0.381 | 0.045 | 0.288 | 0.464 | ＜0.01 | | 0.457 | 0.015 | 0.125 | 0.185 | ＜0.01 |
| SS |  |  |  |  |  | | 0.136 | 0.014 | 0.019 | 0.075 | ＜0.01 |
| Gender | -0.027 | 0.869 | -2.276 | 1.139 | 0.513 | | 0.021 | 0.275 | -0.392 | 0.689 | 0.661 |
| Age | 0.034 | 0.042 | -0.049 | 0.117 | 0.419 | | -0.038 | 0.013 | -0.039 | 0.013 | 0.403 |
| KL | 0.027 | 0.518 | -0.685 | 1.352 | 0.520 | | -0.005 | 0.164 | -0.343 | 0.301 | 0.973 |
| BMI | -0.011 | 0.118 | -0.261 | 0.202 | 0.802 | | -0.032 | 0.037 | -0.105 | 0.042 | 0.388 |
| Bone density | 0.056 | 0.337 | -0.222 | 1.100 | 0.192 | | -0.067 | 0.107 | -0.390 | 0.029 | 0.137 |
| VAS | -0.046 | 0.233 | -0.712 | 0.203 | 0.275 | | 0.012 | 0.074 | -0.122 | 0.168 | 0.878 |
| JLCA | -0.025 | 0.278 | -0.704 | 0.389 | 0.571 | | 0.001 | 0.088 | -0.172 | 0.174 | 0.945 |
| WOMAC | 0.051 | 0.027 | -0.021 | 0.087 | 0.226 | | 0.037 | 0.009 | -0.009 | 0.025 | 0.266 |
| SF-12 | 0.083 | 0.021 | -0.001 | 0.083 | 0.056 | | 0.001 | 0.007 | -0.013 | 0.014 | 0.760 |
| PI | 0.040 | 0.035 | -0.036 | 0.102 | 0.344 | | -0.120 | 0.011 | -0.056 | -0.013 | 0.003 |
| Total effect |  |  |  |  |  | 0.509 | | 0.014 | 0.144 | 0.201 | ＜0.01 |
| Direct effect |  |  |  |  |  | 0.457 | | 0.015 | 0.125 | 0.185 | ＜0.01 |
| Indirect effect |  |  |  |  |  | 0.052 | | 0.018 | 0.019 | 0.088 |  |

Abbreviations, BMI=Body Mass Index, VAS=visual analogue scale, KL=Kellgren-Lawrence, LL=lumbar lordosis, SS=sacral slope, sHKA=sagittal Hip-Knee-Ankle, JLCA=Joint-Line Convergence Angle, PI=Pelvic Incidence, se=Standard error, LLCI=lower level of confidence interval, ULCI=upper level of confidence interval.
